# Development and landscape of maintenance therapy after first-line treatment of advanced or metastatic urothelial carcinoma

**DOI:** 10.3389/fimmu.2025.1541213

**Published:** 2025-07-31

**Authors:** Zengguang Liu, Xiaofeng Cong, Chen Chen, Jiaxin Yin, Ziling Liu

**Affiliations:** Department of Cancer Center, The First Hospital of Jilin University, Changchun, China

**Keywords:** maintenance therapy, urothelial carcinoma (UC), advanced, metastatic, avelumab

## Abstract

Overall survival (OS) in patients with advanced or metastatic urothelial carcinoma (UC) is not optimistic. For a long time, the standard platinum-based chemotherapy has been one of the preferred treatment strategies. Despite the high initial objective response rate (ORR) to first-line chemotherapy in patients with metastatic UC, the rate of achieving complete response (CR) is low, and most patients will relapse within one year after first-line treatment. To further improve the OS of patients with metastatic UC, the success of the CheckMate901 and EV302 studies has brought new therapeutic options for the first-line treatment of these patients. Maintenance or consolidation therapy after first-line treatment is also important to improve the OS of patients with advanced UC. Maintenance therapy after first-line treatment of metastatic UC has undergone a long period of development until the success of the JAVELIN Bladder100 study. For the first time, this study established the application of avelumab as maintenance therapy after first-line platinum-containing chemotherapy. The aim of this paper is to review the development process of avelumab-based maintenance therapy after first-line treatment of advanced or metastatic UC and explore future options for maintenance therapy in patients with advanced or metastatic UC in the light of new first-line treatment options.

## 1 Introduction

As one of the common tumors of the urinary system, bladder cancer ranks the 9th in incidence globally, with more than 610,000 new cases and 220,000 new deaths worldwide in 2022 according to the latest epidemiological data (1). Such a high number of new cases and deaths not only reflects the prevalence of bladder cancer, but also emphasizes the urgent need for more effective prevention, diagnosis, and treatment strategies. Urothelial carcinoma (UC) is the dominant histological subtype of bladder cancer, accounting for about 90% of the bladder cancer cases (2). Understanding the characteristics and biological behavior of UC is critical for developing targeted therapies. At initial presentation, 5% of the diagnosed patients have metastatic disease, with an advanced stage (3). These patients often face a more challenging prognosis than those with earlier stage disease. The presence of metastases means complicating the treatment process and reducing the likelihood of a complete cure. For the initial treatment of advanced UC, platinum-based combination chemotherapy has been the standard of care for patients suitable for platinum-based therapy for decades (4). For patients who are cisplatin-intolerant, the most widely used regimen of gemcitabine in combination with carboplatin also resulted in an ORR of 41.2% and a comparable overall survival (OS) (5). However, although the initial response rate to chemotherapy is high, the rate of achieving CR is low. Most patients will experience disease progression within 9 months of receiving treatment with an OS of 14 to 15 months and the 5-year survival rate for patients with advanced UC is only about 5% (6). These disheartening statistics highlight the unmet medical need in the treatment of advanced or metastatic UC.

To improve the OS of patients with advanced or metastatic UC, the clinical studies of CheckMate901 (7) and EV302 (8) have provided new options for the first-line treatment of advanced UC. However, after receiving first-line treatment, how to maximize the time to disease recurrence and prolong the OS of patients has become a major issue in the first-line treatment of metastatic UC. Maintenance therapy is the continuation of treatment with low-toxicity, high-potency drugs to delay disease recurrence and prolong progression-free survival (PFS) and OS after patients have completed the initial surgical or chemotherapeutic treatment. According to relevant studies, maintenance therapy has been widely used in solid tumors including non-small cell lung cancer (9), ovarian cancer (10), colon cancer (11), and breast cancer (12), greatly improving patients’ OS.

As for advanced or metastatic UC, maintenance therapy has been tried in various forms to improve the OS of the patients until the success of the JAVELIN Bladder100 study (13), which first established the use of avelumab for maintenance therapy in patients with metastatic UC who achieved at least stable disease (SD) after first-line chemotherapy. Compared with the watch-and-wait approach that was the previous standard of care, this sequenced avelumab maintenance therapy after first-line chemotherapy has significantly improved outcomes (14). The CheckMate901 study established the standard regimen of first-line chemotherapy combined with nivolumab treatment and sequential nivolumab maintenance therapy for patients with advanced or metastatic UC (7). The EV302 study successfully identified enfortumab vedotin (EV) combined with pembrolizumab as a new option for standard first-line treatment of patients with advanced UC (8). However, with the results of the CheckMate901 study and the EV302 study suggesting greater success following first-line combination immunotherapy, there does not appear to be a place for the avelumab application for maintenance therapy following first-line treatment in patients treated in this setting.

**Figure 1:**
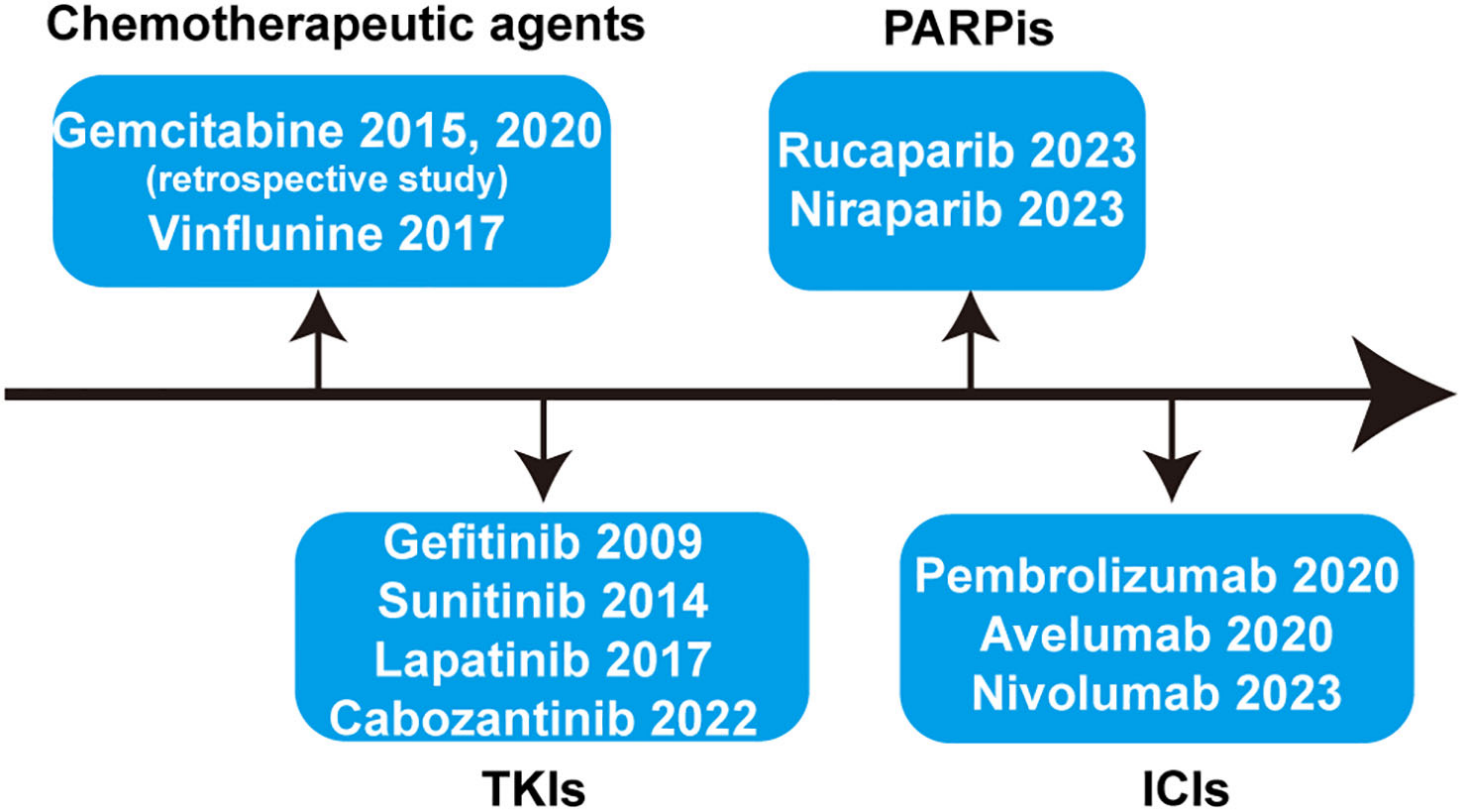
Systematic summary of the drugs applied for maintenance therapy after first-line treatment of advanced or metastatic UC. TKIs, tyrosine kinase inhibitors; PARPis, poly ADP-ribose polymerase inhibitors; ICIs, immune checkpoint inhibitors.

Throughout the development process of first-line treatment for advanced or metastatic UC in the past nearly 40 years, it has mainly experienced the era of chemotherapy, the era of immunotherapy, and the current era of targeted therapy represented by antibody-drug conjugate (ADC) drugs. Due to the high efficiency but short maintenance time of first-line chemotherapy for advanced or metastatic UC and the limitations of subsequent second-line therapy, the relevant research background of the maintenance therapy after first-line treatment is almost always conducted in the context of first-line platinum-based chemotherapy. As shown in **Figure 1**, studies related to maintenance therapy after first-line chemotherapy for advanced or metastatic UC have involved chemotherapeutic agents, tyrosine kinase inhibitors (TKIs), poly ADP-ribose polymerase inhibitors (PARPis), and immune checkpoint inhibitors (ICIs). The aim of this paper is to provide an overview of the maintenance therapy of advanced or metastatic UC and synthesize the development process of avelumab-based maintenance therapy after first-line treatment for patients with advanced UC, while exploring the choice of future maintenance regimens when new first-line treatment options become fully available.

## 2 Switch maintenance therapy with chemotherapeutic agents

With limited options and prior to the introduction of novel agents, maintenance treatment options after first-line chemotherapy for advanced UC are dominated by chemotherapeutic agents. From the current point of view, maintenance therapy with chemotherapy is more like an early application of second-line chemotherapeutic agents. They mainly consist of single-agent chemotherapies with relatively mild side effects and proven efficacy. Gemcitabine and vinflunine are the most widely studied ones. However, the related studies are mainly retrospective.

### 2.1 Gemcitabine

For advanced or metastatic UC, the application of gemcitabine monotherapy provided an ORR of 25% with acceptable adverse effects (15). Here are mainly presented two representative retrospective studies on gemcitabine maintenance after first-line treatment for advanced or metastatic UC. A single-center retrospective study included 66 patients with advanced or metastatic UC whose efficacy was stable after first-line chemotherapy between February 2008 and February 2014. There were 33 patients in the gemcitabine maintenance group and 33 in the observational group. The results showed that the disease-specific survival (DSS) in the maintenance group averaged 15.0 months, which was significantly better than that in the control group (4.0 months) (*p* < 0.001). Multifactorial analysis revealed that visceral metastases, previous chemotherapy efficacy and gemcitabine maintenance therapy were significant prognostic factors for DSS (16). Another single-center retrospective study included 117 patients with advanced or metastatic UC who received 4–6 cycles of gemcitabine combined with cisplatin or carboplatin as first-line chemotherapy from 2014 to 2018. Of these, 58 patients received maintenance therapy with gemcitabine, which demonstrated that gemcitabine maintenance prolonged the median OS by 2.2 months (11.8 vs. 9.6 months, hazard ratio [HR]: 0.621, 95% CI, 0.39-0.97; *p* = 0.026). Grade 3 or higher neutropenia occurred in 17.2% of the patients in the gemcitabine maintenance group (17).

Although these retrospective studies suggest that maintenance with gemcitabine alone after first-line platinum-containing chemotherapy in patients with advanced or metastatic UC is beneficial for survival, large-scale prospective clinical studies are warranted to verify the reliability of this conclusion due to the limitations of the studies.

### 2.2 Vinflunine

Vinflunine monotherapy has been shown to have definite efficacy, a favorable toxicity profile, and no cumulative effects for second-line application in patients with advanced or metastatic UC after platinum-based chemotherapy (18, 19). Based on this, a multicenter, randomized, controlled, open-label, phase 2 trial (MAJA; SOGUG 2011/02) was conducted in Spain aiming to evaluate maintenance therapy with vinflunine + best supportive care (BSC) versus BSC alone in patients with advanced or metastatic UC who had responded after frontline chemotherapy (20). In this trial, 88 patients were enrolled and randomly assigned (1:1) to receive vinflunine [45] or BSC [43] until disease progression. After a median follow-up of 15.6 months, the results indicated that vinflunine maintenance could significantly prolong the median PFS from 4.2 months to 6.5 months (HR: 0.59, 95% CI: 0.37-0.96, *p* = 0.031). Grade 3 or 4 adverse events (AEs) included neutropenia, asthenia or fatigue and constipation but were manageable. However, the use of vinflunine maintenance did not significantly provide a benefit of final OS (16.7 *vs.* 13.2 months, HR: 0.736, 95% CI: 0.44-1.24, *p* = 0.182) (21). Although maintenance therapy with vinflunine provided a PFS benefit in patients with advanced UC or metastatic after first-line chemotherapy, the PFS benefit did not translate into a final OS, which may be an important reason for not being approved as a switch maintenance therapy.

## 3 Switch maintenance therapy with TKIs

### 3.1 Gefitinib

The epidermal growth factor receptor (EGFR) can regulate cell proliferation and apoptosis. Moreover, the expression of EGFR has been observed to be correlated with tumor progression in bladder cancer (22). To systematically evaluate the efficacy of adding gefitinib to platinum-based agents in the first-line treatment of advanced or metastatic UC and the subsequent application of maintenance therapy with gefitinib, a prospective multicenter phase II clinical study (Cancer and Leukemia Group B 90102) was conducted (23). In this trial, 54 eligible patients were enrolled to participate in the study, among whom 25 patients completed the combination therapy and subsequent gefitinib maintenance treatment. Although the new combination therapy was well tolerated and active in advanced transitional carcinoma, the results showed that the median OS and median time to progression were 15.1 months (95% CI: 11.1-21.7 months) and 7.4 months (95% CI: 5.6-9.2 months), respectively, which are similar to the previous findings (6). This suggests that the addition of gefitinib to the platinum-based combination and subsequent gefitinib maintenance therapy did not prolong the median PFS and OS for advanced UC.

### 3.2 Sunitinib

Angiogenesis has been demonstrated to play an important role in the growth and metastasis of UC (24). Additionally, increased vascular endothelial growth factor (VEGF) has been correlated with disease progression in patients with UC (25). Sunitinib is an oral small molecule multi-target TKI including VEGF receptors that has been approved for the treatment of metastatic renal-cell carcinoma (26) and some other solid cancers. Encouraged by this, a randomized, double-blind, prospective phase II trial investigated the efficacy of sunitinib versus placebo for maintenance therapy in patients with advanced or metastatic UC who responded to first-line chemotherapy (27). This trial enrolled 54 patients from November 2006 to January 2011. Due to slow accrual, 26 patients were randomized to the sunitinib maintenance group and 28 patients received placebo. The primary endpoint of the study was the 6-month PFS. At a median follow-up of 10.3 months, there was no difference in the 6-month progression rates between the sunitinib group and placebo group, which were 71.7% (95% CI: 54%-87%) and 64.3% (95% CI: 47%-81%), respectively. Follow-up results of the study indicated that the sunitinib maintenance did not improve the 6-month progression rate, prolong the PFS (2.9 *vs.* 2.7 months) or OS (10.5 *vs*. 10.3 months). Most importantly, sunitinib maintenance resulted in higher rates of AEs, including grade 3–4 thrombocytopenia, diarrhea, mucositis, fatigue and hypertension.

### 3.3 Lapatinib

Lapatinib is an oral small-molecule TKI targeting dual human epidermal growth factor receptor 1 and 2 (HER1 and HER2). Previous studies have shown that EGFR and HER-2 are commonly overexpressed in UC and correlate with prognosis (28). Based on this, Powles et al. initiated a phase III double-blind randomized clinical trial to investigate lapatinib versus placebo as maintenance therapy for patients with HER-1/2-positive metastatic bladder cancer after first-line chemotherapy (29). The primary endpoint of the study was PFS. From 2007 to 2013, a total of 446 patients were screened of which 232 patients with HER-1/2-positive were enrolled and randomly assigned to the lapatinib group and the placebo group. Results showed that lapatinib maintenance therapy did not improve PFS (4.5 *vs.* 5.1 months; HR: 1.07; 95% CI: 0.81-1.43; *p* = 0.63) or OS (12.6 *vs*. 12.0 months; HR: 0.96; 95% CI: 0.70-1.31; *p* = 0.80). The incidence of grade 3 to 4 adverse events (grade 3–4 AEs) was 8.6% and 8.1% in the lapatinib and placebo groups, respectively (*p*= 0.82). Even for patients who were strongly positive for both HER1/2 (3+ on immunohistochemistry; n= 111), the results showed no significant benefit in the lapatinib maintenance group in terms of PFS and OS (*p* > 0. 05). However, given the promising results of HER-2-targeting ADC drugs in advanced UC (30), future exploration of HER-2-targeting ADC drugs in maintenance therapy after first-line treatment of advanced or metastatic UC may also be worthy of further investigation.

### 3.4 Cabozantinib

Cabozantinib is an oral multi-kinase inhibitor targeting MET and VEGF, which has been demonstrated to have activity in patients with metastatic UC (31). A randomized, double-blind, phase II trial evaluated the efficacy of cabozantinib maintenance therapy versus placebo for patients with advanced or metastatic UC who completed 4 to 8 cycles of platinum-based chemotherapy without progression (32). 30 patients were randomized to cabozantinib group and 31 patients were assigned to placebo group from February 2017 to March 2021 and PFS was the primary endpoint. Results of the study revealed that cabozantinib did not significantly prolong PFS (13.7 *vs.* 15.8 weeks, HR: 0.89 favoring cabozantinib, 80% CI: 0.61-1.3, 1-side *p* = 0.35) or OS (HR: 0.80, *p* = 0.35), though with tolerable toxicity profile. The most frequent AEs were fatigue (56.7%), hypertension (43.3%), nausea (30%) and diarrhea (40%).

## 4 Switch maintenance therapy with PARPis

Homologous recombination deficiency (HRD) has been reported in about 10-25% of bladder cancers (33, 34). Analysis of related studies suggests that the high sensitivity of UC to first-line platinum-containing chemotherapy may be due to the underlying HRD (35). Moreover, in addition to sensitivity to platinum, HRD also implies sensitivity to PARP inhibitors. Encouraged by the great success of PARP inhibitors in maintenance therapy for ovarian cancer (10), several studies on PARP inhibitors for maintenance therapy after first-line chemotherapy in advanced or metastatic UC have been gradually conducted.

### 4.1 Rucaparib

Rucaparib is an oral, small molecule inhibitor of PARP. Within the ATLANTIS clinical trial platform, Crabb et al. conducted a randomized, double-blind, biomarker-selected, phase II trial to evaluate the efficacy of maintenance with rucaparib following chemotherapy for patients with advanced or metastatic UC (36). From 24 November 2017 to 2 February 2021, a total of 248 patients were pre-screened for DNA repair deficiency (DRD). 74 patients were positive for DRD and 40 patients were randomly allocated to either the rucaparib group or the placebo group. Results of the study indicated that maintenance therapy with rucaparib could extend PFS (35.3 *vs.* 15.1 weeks, HR: 0.53; 80% CI: 0.30-0.92; one-side *p* = 0.07) but not OS (not reached [NR] *vs.* 72.3 weeks, adjusted HR: 1.22; 80% CI: 0.62-2.38; *p* = 0.35) in DRD biomarker-selected patients with advanced UC. Most of the treatment-related adverse events (TRAEs) were low grade, including fatigue, nausea, rash and raised alanine aminotransferase. However, due to the small sample size in the clinical trial, further development of PARP inhibition maintenance therapy in advanced or metastatic UC is still warranted.

### 4.2 Niraparib

Niraparib is also an oral inhibitor of PARP-1 and PARP-2, which has gained promising results of maintenance therapy in ovarian cancer (37). The Meet-URO12 randomized phase 2 trial systematically evaluated maintenance therapy with the addition of niraparib to BSC in non-biomarker selected patients with advanced or metastatic UC after frontline chemotherapy (38). Between August 2019 and March 2021, 58 patients were randomized (2:1) to niraparib + BSC group (39 patients) and BSC alone group (19 patients). The primary endpoint of the study was PFS. Due to the setting of the approval of maintenance with avelumab in 2020, the study was stopped prematurely. After a median follow-up of 8.5 months, the niraparib maintenance treatment group was not effective in prolonging PFS relative to the BSC group (2.1 *vs.* 2.4 months; HR: 0.92; 95% CI: 0.49-1.75; *p* = 0.81). The main AEs associated with niraparib treatment are anemia, thrombocytopenia, neutropenia, fatigue, mucositis and nausea. Though even in patients with homologous recombination repair (HRR) mutations, the addition of niraparib did not prolong the PFS (2.0 *vs.* 1.9 months), further investigation of PARPi in selected patients with advanced or metastatic UC is still warranted.

## 5 Switch maintenance therapy with ICIs

The emergence of immunotherapy has brought new hope to patients with advanced or metastatic UC. With in-depth research on the immune mechanism of tumor, ICIs represented by anti-PD1/PDL1 inhibitors have gradually become an emerging choice for the treatment of advanced or metastatic UC (39). Based on the success of a series of clinical trials, pembrolizumab, nivolumab, and avelumab have been approved for second-line treatment in patients with advanced or metastatic UC (40–42). It has been suggested that initial chemotherapy may potentially induce immunogenic cell death or lead to depletion of suppressive immune cells, thereby enhancing the therapeutic efficacy of subsequent application of ICIs (43). Despite not all patients could benefit from the ICIs, encouraged by the underlying potential to prolong OS of patients and the potential mechanism, various trials have investigated the efficacy of the application of maintenance therapy with ICIs in patients with advanced or metastatic UC after first-line platinum-based therapy.

### 5.1 Pembrolizumab

Pembrolizumab is an anti-PD1 antibody, which has been widely studied in various solid tumors. An investigator-initiated multicenter double-blind randomized phase II trial conducted by the Hoosier Cancer Research Network (HCRN), namely HCRN GU14–182 trial evaluated the role of maintenance therapy with pembrolizumab in patients with advanced or metastatic UC, who achieved at least stable disease (SD) after up to 8 cycles of first-line combination chemotherapy (44). From 2015 to 2018, a total of 117 patients were screened, and ultimately 108 patients were randomized (1:1) into the pembrolizumab (n = 55) and placebo (n = 53) treatment groups to receive up to 2 years maintenance therapy. The primary objective of the study was PFS, and the secondary endpoints were OS and exploring the correlation between PDL1 expression levels and treatment outcome. At a median follow-up of 12.9 months, a significant prolonged PFS was observed in the pembrolizumab group compared with the placebo group (5.4 *vs.* 3.0 months; HR: 0.65; *p* = 0.04). However, the addition of pembrolizumab did not produce a survival benefit (22 *vs.* 18.7 months; HR: 0.91; 95% CI: 0.52-1.59; *p* = 0.7) and no significant correlation was found between PDL1 expression levels and treatment outcome. The incidence of treatment-related grade 3–4 AEs was 59% versus 38% in the two groups. 20% of the patients in the pembrolizumab cohort underwent steroid therapy for immune-related AEs.

### 5.2 Avelumab

As mentioned before, based on the results of JAVELIN Solid Tumor study (42), avelumab, an anti-PDL1 antibody, has been approved as a second-line choice for patients with advanced or metastatic UC who have progressed after platinum-based chemotherapy upfront (4). JAVELIN Bladder 100, an international, multicenter, randomized, open-label phase 3 trial, further investigated the role of maintenance therapy with avelumab (13). From May 11, 2016 to June 4, 2019, 700 patients with advanced or metastatic UC who achieved at least SD after first-line platinum-based chemotherapy were randomly assigned on a 1:1 basis to receive either avelumab + BSC maintenance therapy or BSC alone, with 51.1% (n = 358) testing positive for PDL1. The primary endpoints of the study were OS in the whole population and OS in the PDL1-expressing population, and the secondary study endpoints were PFS and safety evaluation of the treatment. The results of the study showed that avelumab + BSC significantly prolonged OS (21.4 *vs.* 14.3 months; HR: 0.69; 95% CI: 0.56-0.86; *p* = 0.001) compared with the control group in the overall population, Moreover, avelumab + BSC also provided a survival benefit in the PDL1-positive population (NR *vs.* 17.1 months; HR: 0.56; 95% CI: 0.40-0.79; *p* < 0.001). In addition, the use of avelumab resulted in a benefit in PFS both in the total population (3.7 *vs.* 2.0 months; HR: 0.62; 95% CI: 0.52-0.75) and the PDL1-positive population (5.7 *vs.* 2.1 months; HR: 0.56; 95% CI: 0.43-0.73). The incidence of grade 3 or higher AEs was 47.4% and 25.2% in the treatment and control groups, respectively. 24 patients in the avelumab treatment group reported grade 3 or higher immune-related AEs, and 11.9% of patients discontinuing immunotherapy because of AEs.

The encouraging results of the JAVELIN 100 study directly contributed to the Food and Drug Administration’s (FDA) approval of maintenance therapy with avelumab for a population of patients with advanced or metastatic UC without disease progression after first-line platinum-containing chemotherapy in 2020 (45). Moreover, the results analyzed in most of the subgroups were consistent with the overall population, further supporting that avelumab first-line maintenance therapy can be used in advanced or metastatic UC with different clinical characteristics (46). Long-term follow-up data over more than two years demonstrated that maintenance therapy with avelumab continues to provide a survival benefit (23.8 *vs.* 15.0 months; HR: 0.76; 95% CI: 0.63-0.91; *p* = 0.0036) and the safety profile of the long-term application was consistent with previous reports, with no new safety events, providing more solid evidence for the use of avelumab as a maintenance therapy after first-line chemotherapy for advanced UC (14). Besides, patient-reported outcomes from JAVELIN Bladder 100 revealed that the addition of avelumab as maintenance therapy had less impact on quality of life and improved OS significantly in patients with advanced or metastatic UC (47).

To further explore the potential mechanisms underlying the action of avelumab and to better guide the clinical application, identify the truly beneficial population, and precisely treat the patients, a biomarker analysis of the phase 3 JAVELIN Bladder 100 trial was carried out (48). The authors first analyzed the correlation between PDL1 expression levels and OS benefit, and the results showed that in the high PDL1 expression group, patients treated with Avelumab + BSC had a more significant OS benefit, reducing the risk of death by 65% (HR: 0.35, *p* = 0.0010). Mechanistically, avelumab can restore or enhance the immune response of T cells to tumors by blocking the binding of PD-L1 to PD-1 and CD80 (B7-1), thereby releasing inhibition of T cells. Similarly, an analysis of patients’ tumor mutational burden (TMB) indicated that patients with high TMB had a more significant OS benefit after maintenance therapy with Avelumab + BSC (HR: 0.48, *p* =0.0002). Gene expression analysis revealed that the avelumab might prolong the OS by regulating several immune-related gene sets representing innate immunity (such as natural killer (NK) cells, macrophages, and dendritic cells) as well as adaptive immunity (B cells, CD4 T cells, and CD8 T cells). In addition to this, considering the molecular complexity and tumor heterogeneity of advanced or metastatic UC and the fact that OS results of avelumab + BSC may be associated with many genes representing multiple biological pathways, the authors constructed two multiparametric models, gene expression/mutation model (GEM model) and a combination of clinical, molecular and cellular model (CMC model). The application of both models showed that they could identify patients with prolonged OS in the Avelumab + BSC group, but could not identify patients with prolonged OS in the BSC group, characterizing them as predictive rather than prognostic (48). Nevertheless, prospective validation of these potential biomarkers is required to better guide clinical decision-making. Moreover, the complex biologic pathways underlying the survival benefit of ICIs inhibition in patients with advanced or metastatic UC suggest that multiple biomarkers may be needed to identify patients who will benefit from treatment.

Besides, considering the molecular complexity and tumor heterogeneity of advanced or metastatic UC, another recent study published in *Cancer Cell* identified four molecular subtypes of UC by integrating molecular data from patients with UC and the results of a randomized clinical trial: cluster NMF1 luminal desert, cluster NMF2 stromal, cluster NMF3 immune, and cluster NMF4 basal (49). These findings offer new perspectives for the precision treatment of UC, suggesting that customizing treatment regimens based on molecular subtypes may contribute to improving treatment outcomes. However, future studies are needed to further prospectively validate the classification of these molecular subtypes and explore additional biomarkers and therapeutic targets for more personalized medical strategies.

### 5.3 Nivolumab

Nivolumab, as an anti-PD1 antibody, has also been approved as a choice for the second-line therapy of metastatic UC based on the results of CheckMate275 (41). CheckMate901 trial assessed the role of maintenance therapy with nivolumab when patients were received the first-line therapy with nivolumab + gemcitabine-cisplatin (7). The CheckMate901 study is an international, multicenter, open-label, phase III clinical study in which 608 patients with previously untreated metastatic UC were randomly assigned 1:1 to receive either gemcitabine in combination with cisplatin (up to 6 cycles) or gemcitabine in combination with cisplatin + nivolumab (up to 6 cycles), followed by subsequent application of maintenance therapy with nivolumab for up to 2 years. OS and PFS were the coprimary endpoints. At a median follow-up of 33.6 months, we could find that the combination of concurrent immunotherapy + chemotherapy combinations followed by sequential nivolumab monotherapy maintenance therapy significantly brought about a prolongation of PFS (7.9 *vs.* 7.6 months; HR: 0.72; 95% CI: 0.59-0.88; *p* = 0.001) and OS (21.7 *vs.* 18.9 months; HR: 0.78; 95% CI: 0.63-0.96; *p* = 0.02). The rates of grade 3 or higher AEs were 61.8% and 51.7% in the combination group relative to the chemotherapy group, and the addition of nivolumab did not result in new safety events. Based on the successful results of CheckMate901, the combination of gemcitabine + cisplatin + nivolumab followed by nivolumab maintenance therapy has become one of the standard regimens for first-line treatment of untreated advanced or metastatic UC.

## 6 Emerging explorations and future prospect for maintenance therapy

Our study systematically reviewed the history of the development of maintenance therapy after first-line treatment in patients with advanced or metastatic UC (**Supplementary Table S1**). For patients with advanced or metastatic UC, maintenance therapy with avelumab + BSC is the standard of care as defined by current guidelines in the window of non-progression after first-line platinum-containing two-agent chemotherapy (4, 50–52) (**Table 1**). Most of the current and future investigational studies exploring new modalities of maintenance therapy for advanced or metastatic UC are based on the solid efficacy of avelumab. The success of CheckMate901 study and EV302 study have provided new options for patients with advanced or metastatic UC and have been identified to significantly prolong the PFS and OS. On the one hand, the success of the CheckMate901 study directly establishes a strategy for the subsequent application of nivolumab maintenance therapy in patients with advanced or metastatic UC in the context of first-line platinum-containing doublet chemotherapy combined with nivolumab for those with at least stable disease; on the other hand, according to the design of the EV302 study, we can learn that although the study specifies a maximum application duration of 2 years for pembrolizumab, the administration of EV is continued until intolerable toxicity or disease progression. This dosing regimen is equivalent to the disguised use of EV as maintenance therapy following the combination of EV and pembrolizumab. Although the EV302 and CheckMate901 studies have identified new modalities of first-line treatment for patients with metastatic UC, the context of research on maintenance therapy in future remains predominantly non-progressing disease after first-line platinum-based chemotherapy.

**Table 1:** Summary of maintenance therapy recommendation in advanced or metastatic UC of the guidelines.

Platinum Eligibility Status	First-line therapy	Maintenance therapy	NCCN (4) guidelines	ESMO (50) guidelines	EAU (51) guidelines
Cisplatin eligible	Pem + EV	none	–	–	–
	G + P	Avelumab	✓	✓	✓
	Nivo + G + P	Nivolumab	✓	✓	✓
	DDMVAC with growth factor support	Avelumab	✓	none	none
Cisplatin ineligible	Pem + EV	none	–	–	–
	G + C	Avelumab	✓	✓	none

Pem, prolizumab; EV, enfortumab vedotin; G, gemcitabine; P, cisplatin; Nivo, nivolumab;

DDMVAC, methotrexate, vinblastine, doxorubicin, and cisplatin; C, carboplatin; NCCN, National Comprehensive Cancer Network; ESMO, European Society for Medical Oncology; EAU, European Association of Urology.

By searching in the Web of Clinical Trials (https://clinicaltrials.gov/), we identified four relevant trials exploring the maintenance therapy of advanced UC. The ongoing TALASUR study (NCT04678362) is a single-arm phase II study to explore the efficacy of avelumab in combination with talazoparib (an orally PARP inhibitor) for maintenance therapy after first-line chemotherapy in advanced UC. The study proposes to enroll 50 subjects and the primary study endpoint is PFS. The MAIN-CAV study (NCT05092958) is an ongoing randomized, multicenter, open labeled, phase III study based on the combination of cabozantinib and avelumab. The study estimates to enroll 654 patients and aims to investigate whether the addition of cabozantinib to avelumab versus avelumab alone will prolong OS in patients with metastatic UC who did not progress during first-line platinum-containing chemotherapy. In addition to this, another ongoing randomized, open-labeled phase II study, the JAVELIN Bladder Medley study (NCT05327530), aims to explore the safety and efficacy of multiple avelumab-based combination modalities for maintenance therapy in patients with advanced UC whose disease did not progress following first-line platinum-containing chemotherapy. 256 participants will participate in the trial and randomly assigned to four groups: group A: avelumab; group B: avelumab + sacituzumab govitecan; group C: avelumab + M6223 (anti-T cell-immuno-receptor with Ig and ITM domains [anti-TIGIT]); group D: avelumab + NKTR-255 (a novel polymer-conjugated recombinant human interlukin-15). The primary objectives are PFS and the safety associated with the treatment. Another ongoing international, phase II trial (VEXILLUM, NCT05219435) assesses the efficacy of maintenance therapy with nivolumab + ipilimumab in patients with unresectable advanced UC that did not progress during or following completion of first-line chemotherapy. 66 patients will be enrolled in this trial and the primary outcome measures are PFS and PFS in PDL1 positive patients. **Table 2** mainly summarizes the important ongoing and the future maintenance therapy trials in unresectable advanced or metastatic UC.

**Table 2:** Summary of the ongoing maintenance therapy clinical trials of advanced or metastatic UC.

Clinical trial	Phase	Enrollment patients (N)	Treatment arms	Primary endpoint	Estimated completion date
TALASUR study (NCT04678362)	II	50	Avelumab + Talazoparib	PFS	December 2025
MAIN-CAV study (NCT05092958)	III	654	Avelumab + cabozantinib *vs.* Avelumab	OS	December 2024
JAVELIN Bladder Medley study (NCT05327530)	II	256	A: Avelumab B: Avelumab + sactuzumab govitecan C: Avelumab + M6223 D: Avelumab + NKTR-255	PFS and Safety	January 2025
VEXILLUM study (NCT05219435)	II	66	Nivolumab + Ipilimumab	PFS and PFS in PDL1 (+) patients	March 2025

## 7 Conclusion

Although the pattern of first-line treatment for advanced or metastatic UC has gradually changed, platinum-containing dual-agent chemotherapy is still one of the effective choices for the first-line treatment of advanced or metastatic UC. Based on current research, there is still broad room for exploration in the future regarding maintenance therapy after platinum-containing dual-agent chemotherapy.

In terms of drug research and development, one of the key directions is to continue to explore new immunotherapy drugs and targeted drugs. For example, studies have been conducted to explore the efficacy of new antibody-coupled drugs in UC (such as ADC drugs targeting Trop2 and HER-2), which provide new options for future treatment and is expected to further improve patient outcomes. In addition, the optimization of combination therapy strategies is also a focus of future research. The combination of different ICIs with various ADC drugs, as well as the combination of ADC drugs targeting different targets are all directions worth exploring in the future. Besides, precision medicine has great potential in the treatment of advanced or metastatic UC. Considering the individual differences of patients, such as the impact of PDL1 expression level, tumor mutation load, HRR mutation, expression of the FGFR2/3 genes and other factors on the therapeutic effect, more precise treatment plans can be formulated for patients with advanced or metastatic UC. The relevance and effectiveness of treatment can be improved by delving into the relationship between these factors and different treatments.

In conclusion, in the future, in the field of maintenance therapy after first-line treatment of advanced or metastatic UC, in-depth research on drug development, optimization of combination therapy strategies and precision medicine is expected to bring better treatment results and survival hope for patients with advanced or metastatic UC.
