# Supplementary material for: Development and landscape of maintenance therapy after first-line treatment of advanced or metastatic urothelial carcinoma
Source: Front Immunol. 2025 Jul 31;16:1541213. doi: 10.3389/fimmu.2025.1541213 (PMC12350496; doi:10.3389/fimmu.2025.1541213)
Supplement: Supplementary file 1 [file Table1.docx]

Supplementary Material

# Supplementary Table

**Table S1.** Systematic summary of the clinical trials for maintenance therapy in advanced or metastatic UC

|  | **Chemo** | **TKIs** | | | | **PARPis** | | **ICIs** | | |
| --- | --- | --- | --- | --- | --- | --- | --- | --- | --- | --- |
| Published year | 2017 (20)  2020 (21) | 2009 (23) | 2014 (27) | 2017 (29) | 2022 (32) | 2022 (36) | 2023 (38) | 2020  (44) | 2020 (13)  2023 (14) | 2023 (7) |
| Study | MAJA; SOGUG 2011/02 | Cancer and Leukemia Group B 90102 | NCT00393796 | UK NCRI LAMB | ATLANTIS | ATLANTIS | Meet-URO12 | Hoosier Cancer Research Network GU14-182 | JAVELIN Bladder 100 | CheckMate 901 |
| Intervention | Vinflunine  *vs.*  BSC | Gefitinib + chemo and maintenance with gefitinib | Sunitinib  *vs.*  Placebo | Lapatinib  *vs.*  Placebo | Cabozantinib  *vs.*  Placebo | Rucaparib  *vs.*  Placebo | Niraparib +BSC  *vs.*  BSC | Pembrolizumab  *vs.*  Placebo | Avelumab + BSC  *vs.*  BSC | Nivo + chemo and maintenance with nivo *vs.* chemo |
| Phase | II | II | II | III | II | II | II | II | III | III |
| Enrolled patients | 88  (45 *vs.* 43) | 54 | 54  (26 *vs.*28) | 232  (116 *vs*. 116) | 61  (31 *vs.* 30) | 40  (20 *vs.*20) | 58 (2:1)  (39 *vs.* 19) | 108  (55 *vs.*53) | 700 (350 *vs.* 350) | 608 (304 *vs.* 304) |
| Median follow up (months) | 15.6 | 39.5 | 10.3 | Not known | Not known | 23.65 | 8.5 | 12.9 | 19 | 33.6 |
| Primary endpoint | Median  PFS > 5.3 months | ORR | 6-month  progression rate | PFS | PFS | PFS | PFS | PFS | OS | OS and PFS |
| ORR (%) | 21 *vs.* 7 | 42.6 | Not known | 14 *vs.* 8 | Not known | Not known | Not known | 23 *vs.* 10 | 9.7 *vs.* 1.4 | 57.6 *vs.*43.1 |
| OS  (months) | 16.7 *vs.* 13.2; *p* = 0.182 | 15.1 | 10.5 *vs.* 10.3 | 12.6 *vs.* 12.0;  *p* = 0.80 | 18.8 *vs.* 20.7;  *p* = 0.25 | NR *vs.* 18.08;  *p* = 0.35 | Not known | 22 *vs.* 18.7;  *p* = 0.7 | 21.4 *vs.* 14.3;  *p* = 0.001 | 21.7 *vs*. 18.9; *p* = 0.02 |
| PFS (months) | 6.5 *vs.* 4.2;  *p* = 0.031 | 7.4 | 2.9 *vs.* 2.7 | 4.5 *vs.* 5.1;  *p* = 0.63 | 3.425 *vs.* 3.95;  *p* = 0.35 | 8.825 *vs.* 3.775;  *p* = 0.07 | 2.1 *vs.* 2.4; *p* = 0.81 | 5.4 *vs*. 3.0;  *p* = 0.04 | 3.7 *vs* 2.0 | 7.9 *vs.* 7.6;  *p* = 0.001 |
| Grade 3-4 AEs | 92% | 22% | Thrombocytopenia (23.1)  Diarrhea and mucositis (15.4)  Fatigue (15.4)  Hypertension (11.5) | 8.6% | Not known | 5.3% | 66% *vs.* 16% | 59% *vs.* 38% | 47.4% *vs.* 25.2% | 61.8% *vs.* 51.7% |
| Drug Discontinuation rate due to drug-related AEs | 7% | Not known | 26.9% | 6% *vs.* 5% | Not known | 26.3%  *vs.* 5% | Not known | 17% | 11.9% *vs.* 0.6% | 21.1% *vs.*17.4% |
| Study Outcomes | Prolonged PFS | No OS improvement | Study closed | No PFS improvement | No PFS improvement | Extended PFS in biomarker-selected patients | No PFS improvement | Prolonged PFS | Prolonged PFS and OS | Prolonged PFS and OS |

Chemo: chemotherapy; TKIs: tyrosine kinase inhibitors; PARPis: poly ADP-ribose polymerase inhibitors; ICIs: immune checkpoint inhibitors; BSC: best support care; Nivo: nivolumab; OS: overall survival; PFS: progression free time; ORR: objective response rate; NR: not reached; AEs: adverse events.

**
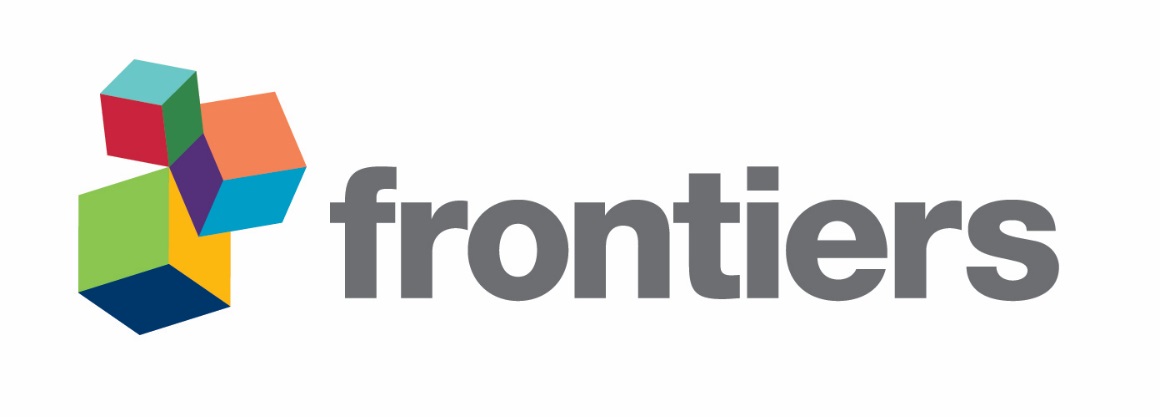
**
